# Supplementary material for: End Sequence Analysis Toolkit (ESAT) expands the extractable information from single-cell RNA-seq data
Source: Genome Res. 2016 Oct;26(10):1397–410. doi: 10.1101/gr.207902.116 (PMC5052061; doi:10.1101/gr.207902.116)
Supplement: Supplemental Material [file supp_gr.207902.116_Supp_Materials.tar.gz › ESAT-esat_v0.1/doc/html/classbroad_1_1core_1_1siphy_1_1_tree_scaler_i_o-members.html]

ESAT: Member List


ESAT


- Main Page
- Related Pages
- Packages
- Classes
- Files

- Class List
- Class Index
- Class Hierarchy
- Class Members

All Classes Namespaces Files Functions Variables Enumerator Pages

- broad
- core
- siphy
- TreeScalerIO

broad.core.siphy.TreeScalerIO Member List

This is the complete list of members for broad.core.siphy.TreeScalerIO, including all inherited members.

|  |  |  |
| --- | --- | --- |
| addListener(ScaledWindowParserListener listener) | broad.core.siphy.TreeScalerIO |  |
| addWindow(ScaledWindow sw) | broad.core.siphy.TreeScalerIO |  |
| clear() | broad.core.siphy.TreeScalerIO |  |
| getEstimationWindow() | broad.core.siphy.TreeScalerIO |  |
| getScaledWindows() | broad.core.siphy.TreeScalerIO |  |
| getTotalInSources() | broad.core.siphy.TreeScalerIO |  |
| load(InputStream is, int shift, String chr) | broad.core.siphy.TreeScalerIO |  |
| load(File source, int shift, String chr) | broad.core.siphy.TreeScalerIO |  |
| load(InputStream is) | broad.core.siphy.TreeScalerIO |  |
| loadAtMostRandom(File source, String chr, int maxNeutralOmegas) | broad.core.siphy.TreeScalerIO |  |
| loadIntegrated(File source, double minPVal) | broad.core.siphy.TreeScalerIO |  |
| loadIntoDistribution(File source, EmpiricalDistribution ed, boolean shortFormat) | broad.core.siphy.TreeScalerIO |  |
| loadOldIntegrated(File source, int shift, String chr, double minPVal) | broad.core.siphy.TreeScalerIO |  |
| parse(File source, int shift, String chr) | broad.core.siphy.TreeScalerIO |  |
| permute() | broad.core.siphy.TreeScalerIO |  |
| setEstimationWindow(int estimationWindow) | broad.core.siphy.TreeScalerIO |  |
| setNumberFormatter(DecimalFormat formatter) | broad.core.siphy.TreeScalerIO |  |
| stitch(int window, int skip) | broad.core.siphy.TreeScalerIO |  |
| TreeScalerIO(int windowSize) | broad.core.siphy.TreeScalerIO |  |
| TreeScalerIO() | broad.core.siphy.TreeScalerIO |  |
| writeFull(BufferedWriter bw, List< ScaledWindow > windows) | broad.core.siphy.TreeScalerIO |  |
| writeFull(BufferedWriter bw) | broad.core.siphy.TreeScalerIO |  |
| writeFullAsGenomeGraphUsingLogPval(BufferedWriter bw) | broad.core.siphy.TreeScalerIO |  |


---

Generated on Fri Aug 15 2014 15:10:57 for ESAT by  

 1.8.7
